# The Register

**Published:** 1849-07

**Authors:** 


### THE REGISTER.

This number closes the second volume of this work, and we would remark that we have spent much time and labor in bringing it to its present condition. The first volume was urged through the press by a special contract, which we felt not at liberty to withdraw from, as it was entered into by the advice of the Society. Had such not been the case, it should never have gone out in such a dress. The second volume speaks for itself. Yet several improvements might be made if the profession would take that interest in the advance of dental literature which it should. We have now been two years connected with this work as one of its editors; long enough to learn that those most liberal in promises, are most stingy in performance.

Our colleague formerly of St. Louis, owing to his removal to California, cannot longer act as one of its editors. We have received from him efficient aid. Other duties press upon us so much of late, that we feel the Society would do us quite a favor, to select some one else to attend to the Register.—[CIN. ED.

